# Similar efficacy, safety and immunogenicity of adalimumab biosimilar BI 695501 and Humira reference product in patients with moderately to severely active rheumatoid arthritis: results from the phase III randomised VOLTAIRE-RA equivalence study

**DOI:** 10.1136/annrheumdis-2017-212245

**Published:** 2018-03-07

**Authors:** Stanley B Cohen, Alberto Alonso-Ruiz, Piotr A Klimiuk, Eric C Lee, Nuala Peter, Ivo Sonderegger, Deepak Assudani

**Affiliations:** 1 Metroplex Clinical Research Center, Dallas, Texas, USA; 2 Hospital de Cruces, Barakaldo, Spain; 3 Medical University of Bialystok, Bialystok, Poland; 4 Inland Rheumatology, Upland, California, USA; 5 Boehringer Ingelheim, Ingelheim am Rhein, Germany

**Keywords:** rheumatoid arthritis, anti-tnf, autoimmune diseases, dmards (biologic), treatment

## Abstract

**Objective:**

To demonstrate clinical equivalence of adalimumab biosimilar candidate BI 695501 with Humira.

**Methods:**

Patients with active rheumatoid arthritis on stable methotrexate were randomised to BI 695501 or Humira in a double-blind, parallel-group, equivalence study. At week 24, patients were rerandomised to continue BI 695501 or Humira, or switch from Humira to BI 695501. The coprimary endpoints were the percentage of patients achieving the American College of Rheumatology 20% response criteria (ACR20) at weeks 12 and 24. Further efficacy and safety endpoints and immunogenicity were assessed up to week 58.

**Results:**

645 patients were randomised. At week 12, 67.0% and 61.1% (90% CI –0.9 to 12.7) of patients receiving BI 695501 (n=324) and Humira (n=321), respectively, achieved ACR20; at week 24 the corresponding values were 69.0% and 64.5% (95% CI –3.4 to 12.5). These differences were within prespecified margins (week 12: 90% CI (–12% to 15%); week 24: 95% CI (−15% to 15%)), demonstrating therapeutic bioequivalence. 593 patients were rerandomised at week 24. Up to week 48, mean change from baseline in Disease Activity Score 28-erythrocyte sedimentation rate and ACR20/ACR50/ACR70 response rates were similar across the switched (n=147), continuous BI 695501 (n=298) and continuous Humira (n=148) groups. Similar immunogenicity (antidrug antibodies (ADAs), ADA titres and neutralising antibodies) was seen between BI 695501 and Humira (to week 24) and across rerandomised groups (to week 48). Safety and tolerability profiles were similar between groups.

**Conclusions:**

BI 695501 demonstrated similar efficacy, safety and immunogenicity to Humira; switch from Humira to BI 695501 had no impact on efficacy, safety and immunogenicity.

**Trial registration number:**

NCT02137226, Results.

## Introduction

Biosimilars are reproductions of existing biologic molecules that have a high degree of similarity to their reference products, including their molecular structure, biological function and effect in patients, that is, efficacy, safety and immunogenicity. Development programmes for biosimilars are specifically designed to demonstrate similarity to the reference product^1^; they do not assess efficacy and safety profiles versus a current standard of care. These requirements, defined by the Food and Drug Administration (FDA)^1^ and European Medicines Agency (EMA),^2^ include a phase III clinical trial comparing clinical efficacy and safety of the biosimilar with its reference product in a clinical model that is sensitive to detect any potential clinically meaningful differences between the two versions of the molecule.^1 3^

The wide use of biologics across a number of diseases has led to significant improvements in patients’ health. This has come with an increase in healthcare expenditure.^4^ However, the advent of biosimilars to infliximab, etanercept and rituximab has introduced more treatment choice^5^ and led to cost reductions.

The tumour necrosis factor inhibitor Humira (adalimumab, AbbVie) is an established biologic treatment for a number of immune-mediated inflammatory diseases, including rheumatoid arthritis (RA), psoriatic arthritis and inflammatory bowel disease. A number of biosimilar candidates to Humira are currently in development, including the recently approved BI 695501 (Cyltezo, adalimumab-adbm, Boehringer Ingelheim).^6^ Extensive comparison of the physicochemical structure and biologic function of BI 695501 and Humira showed structural similarity and comparable functionality (Sonderegger I, Wittner M, 2018. Manuscripts in preparation). Furthermore, the VOLTAIRE-PK study (NCT02045979) established three-way pharmacokinetic similarity between BI 695501, and European Union (EU)-approved and USA-approved Humira.^6^

The VOLTAIRE-RA trial constituted the final step of the biosimilarity assessment for BI 695501.

## Methods

### Study design

**Figure 1:**
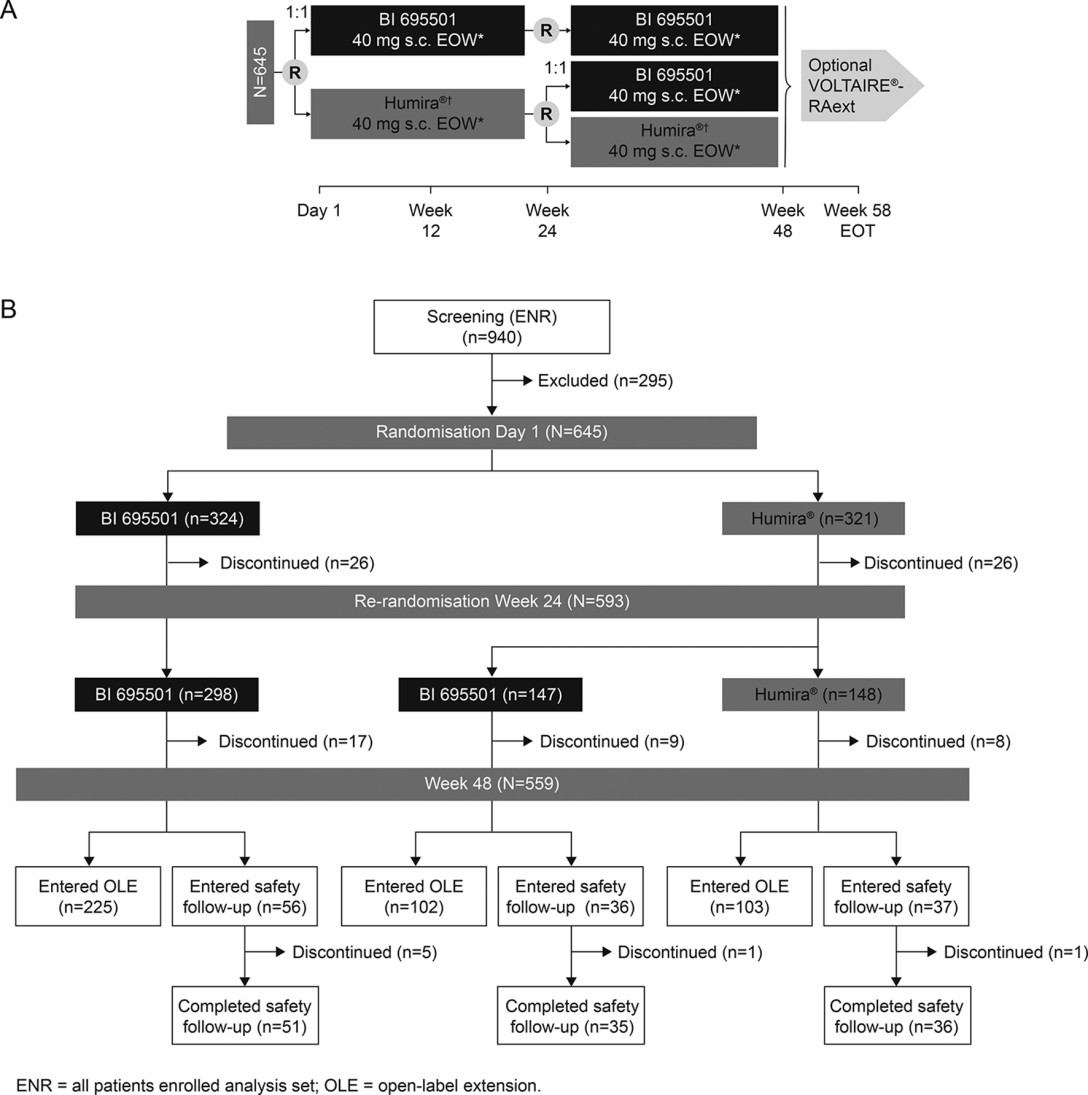
VOLTAIRE-RA study design (A) and patient disposition (B). *Patients continued with methotrexate 15–25 mg/week. Methotrexate 10–14 mg/week was permitted for patients with documented intolerance to higher doses of methotrexate. †Humira 40 mg/0.8 mL solution for subcutaneous injection. EOT, end of treatment; EOW, every other week; n, number of patients per group.

VOLTAIRE-RA was a randomised, double-blind, parallel-arm, 58-week equivalence trial of BI 695501 and USA-sourced Humira (NCT02137226; figure 1A) in 14 countries (115 sites). Patients with moderate-to-severe RA on stable methotrexate (MTX) were randomised 1:1 to receive BI 695501 or Humira 40 mg subcutaneously by prefilled syringe once every 2 weeks for 24 weeks by suitably qualified, designated blinded trial personnel either on-site or at the patient’s home. First doses of trial medication were administered at the site. Randomisation (via an interactive response technology system; Almac Clinical Technologies, Souderton, Pennsylvania, USA) included stratification according to region (Asia, EU, Latin America, USA) and prior exposure to a biologic agent (yes/no) (see online supplementary appendix A for further details). Patients originally randomised to Humira were rerandomised at week 24 to either continue Humira (continuous Humira) or transition to BI 695501 (Humira to BI 695501). Patients originally randomised to BI 695501 were dummy-rerandomised to continue BI 695501 (continuous BI 695501). Rerandomisation was stratified by prior exposure to a biologic agent only.

10.1136/annrheumdis-2017-212245.supp1 Supplementary data

At the end of the trial, qualifying patients could enter an open-label extension (OLE; VOLTAIRE-RAext; NCT02640612), where all patients received BI 695501 for ≤48 weeks; otherwise, safety follow-up occurred at week 58. The study was conducted in accordance with the Declaration of Helsinki. All patients provided written informed consent.

### Objectives and endpoints

The primary objective of the study was to demonstrate equivalent clinical efficacy of BI 695501 and Humira. Coprimary endpoints were the percentage of patients achieving the American College of Rheumatology 20% response criteria (ACR20) at week 12 (requested by FDA) and week 24 (requested by EMA). Prespecified secondary endpoints were change from baseline in Disease Activity Score in 28 joints (DAS28) using erythrocyte sedimentation rate (ESR) at weeks 12 and 24, and the percentage of patients with drug-related treatment-emergent adverse events (AEs). Further endpoints included ACR-based and DAS-based parameters at various time points, quality of life (36-Item Short-Form Health Survey (SF-36) V.2), AEs (including infections/serious infections, hypersensitivity reactions, drug-induced liver injury, injection site reactions) and immunogenicity (antidrug antibodies (ADAs), neutralising antibodies (nAbs), drug levels).

### Patients

Adults (18–80 years) with moderately to severely active RA for ≥6 months, defined by ≥6 swollen joints (66 joint count) and ≥6 tender joints (68 joint count), at screening and baseline, and either ESR >28 mm/hour or C reactive protein (CRP) >1.0 mg/dL at screening, were enrolled. Patients must have received 15–25 mg/week MTX background treatment for ≥12 weeks prior to enrolment. MTX 10–14 mg/week was permitted for patients intolerant to higher doses. Patients could have been on oral corticosteroids ≤10 mg/day prednisolone or equivalent (stable for 4 weeks prior to day 1) and stable non-steroidal anti-inflammatory drugs for 2 weeks prior to day 1.

Exclusion criteria included previous RA treatment with adalimumab or >1 other biologic, active infection, hypersensitivity reactions or AEs to agents similar to the study drugs or their excipients (full criteria available in online supplementary appendix B).

### Statistical analyses

For determination of the primary endpoint, non-responder imputation was used for patients who discontinued prior to that time point. For patients who had not discontinued but had missing data, multiple imputation was used. At each time point (weeks 12 and 24), and on each of the complete data sets following the imputation, logistic regression was applied, including fixed, categorical effects of treatment and prior exposure to a biologic agent (yes/no), and continuous effects of baseline DAS28-ESR. The multiple risk differences and CIs on the individual complete data sets were calculated using the Reeve method,^7^ and combined using Rubin’s rules.^8^

Region was not included in the model due to sparse data in some regions. This was known shortly after final recruitment and included in a protocol amendment prior to database lock.

The primary endpoint, analysed as described above and based on the full analysis set (FAS), was met if the upper and lower CIs of both coprimary endpoints were contained within the prespecified margins. Equivalence was achieved when the difference in ACR20 response rates (BI 695501 minus Humira) was within −12% and 15% (90% CI; week 12 per FDA consultation) and within −15% and 15% (95% CI; week 24 per EMA consultation). An FDA-agreed asymmetrical margin at week 12 was defined, with a slightly higher upper bound of +15% to allow for variations in techniques and response rates used in the calculation of the margins. For this test to be performed with adequate power (86%–91%), a sample size of ~650 patients was needed (FAS). This sample size was based on an assumed treatment difference in ACR20 response rates of 0%, a standard proportion of 59% and an asymmetrical equivalence margin of (−12% to 15%) at week 12, with corresponding values of 0%, 63% (−15% to 15%) at week 24.

The FAS contained all patients who received at least one dose of trial drug and who had all measures required for the efficacy endpoints (ACR20 at weeks 12 and 24) at baseline and at least once postbaseline. The per-protocol analysis set (PPS) contained all patients in the FAS who did not experience any important protocol deviations relevant for efficacy (eg, severe deviation to the restricted disease-modifying anti-rheumatic drug (DMARD) therapy prior to primary endpoint assessment). The safety analysis set (SAF) contained all patients who received at least one dose of trial drug. Descriptive safety data were coded according to MedDRA V.19.0. Data were analysed using SAS software Version 5.0.

The secondary efficacy endpoint of change from baseline in DAS28-ESR was assessed via an analysis of covariance model, using multiple imputation method for missing data. ACR20 at week 48, ACR50 and ACR70 and further efficacy endpoints were computed using the same missing data methodology. Exploratory endpoints were analysed by descriptive statistical methods.

### Immunogenicity

Immunogenicity evaluations were performed (SAF) as previously described (overview available in online supplementary appendix C).^6^

## Results

### Patient disposition and baseline characteristics

The first patient was screened on 4 February 2015. Across 137 centres, 645 patients were randomised (3 March 2015–18 October 2015) 1:1 to BI 695501 (n=324) and Humira (n=321) (SAF). Six patients were excluded from the FAS (lack of postbaseline efficacy assessment); 38 patients were excluded from the PPS (protocol deviations). At week 24, 593 patients were rerandomised to continuous BI 695501 (n=298), continuous Humira (n=148) and Humira to BI 695501 (n=147); 85 (13.2%) patients discontinued the trial prematurely. Last patient, last visit occurred on 18 October 2016. There were no differences in the rate of treatment or trial discontinuation between treatment groups. Patient disposition and geographical distribution are presented in figure 1B and online supplementary table S1, respectively.

Baseline demographics and clinical characteristics were balanced between treatment groups (table 1).

**Table 1:** Baseline demographics and clinical characteristics (SAF)

Characteristics (unit)	BI 695501 (n=324)	Humira (n=321)
Mean age, years (SD)	53.7 (12.0)	53.6 (11.3)
Age, n (%)		
<65 years	264 (81.5)	275 (85.7)
≥65 years	60 (18.5)	46 (14.3)
Women, n (%)	267 (82.4)	269 (83.8)
Mean weight, kg (SD)	73.1 (16.9)	75.1 (17.1)
Mean BMI, kg/m^2^ (SD)	27.0 (5.4)	27.9 (6.3)
Race, n (%)		
Asian	8 (2.5)	6 (1.9)
Black or African–American	6 (1.9)	7 (2.2)
White	309 (95.4)	304 (94.7)
Other*	1 (0.3)	4 (1.2)
Ethnicity, n (%)		
Hispanic or Latino	45 (13.9)	44 (13.7)
Not Hispanic or Latino	274 (84.6)	276 (86.0)
Not reported	5 (1.5)	1 (0.3)
Geographical region, n (%)		
Asia	6 (1.9)	6 (1.9)
Europe	231 (71.3)	228 (71.0)
Latin America	25 (7.7)	26 (8.1)
USA	62 (19.1)	61 (19.0)
Mean duration of RA, years (SD)	7.3 (7.2)	7.0 (6.8)
Duration of RA category, n (%)		
<2 years	87 (26.9)	76 (23.7)
≥2 years	234 (72.2)	238 (74.1)
Missing	3 (0.9)	7 (2.2)
Patients with autoantibodies, n (%)		
RF-positive	281 (86.7)	281 (87.5)
Anti-CCP positive	218 (67.3)	237 (73.8)
DAS28 and components		
DAS28-ESR, mean (SD)	6.6 (0.8)	6.6 (0.8)
ESR, mm/hour, mean (SD)	45.5 (19.2)	43.2 (18.0)
HAQ-DI, median (IQR)	1.5 (0.8)	1.5 (0.9)
Swollen joint count, mean (SD)	17.1 (10.4)	15.9 (9.1)
Tender joint count, mean (SD)	25.3 (13.7)	24.9 (13.3)
Prior exposure to a biological† agent, n (%)		
Yes	85 (26.2)	86 (26.8)
No	239 (73.8)	235 (73.2)
Prior cDMARD‡ therapies, mean (SD)	2.2 (1.4)	2.4 (1.5)
Mean MTX dose, mg/week (SD)	16.3 (3.6)	16.8 (3.9)
Patients with ADAs, n (%)		
ADA-positive	11 (3.4)	21 (6.5)
Neutralising ADA-positive	9 (2.8)	16 (5.0)

*Not American Indian or Alaska Native, or Native Hawaiian or Other Pacific Islander.

†Prior biologics included etanercept, tocilizumab, infliximab, certolizumab, rituximab, abatacept and golimumab.

‡Prior cDMARDs included methotrexate, sulfasalazine, leflunomide, chloroquine, hydroxychloroquine and gold.

ADA, antidrug antibody; BMI, body mass index; CCP, cyclic citrullinated peptide; cDMARD, conventional disease-modifying anti-rheumatic drug; DAS28, Disease Activity Score 28-joint count; ESR, erythrocyte sedimentation rate; HAQ-DI, Health Assessment Questionnaire-Disability Index; MTX, methotrexate; n, number of patients per group; RA, rheumatoid arthritis; RF, rheumatoid factor; SAF, safety analysis set.

### Efficacy

#### Results at week 24

Both coprimary endpoints met the predefined criteria, demonstrating therapeutic equivalence of BI 695501 and Humira at weeks 12 and 24 (table 2). The difference in the proportion of patients achieving an ACR20 response was within the prespecified interval at week 12 (90% CI −0.9 to 12.7) and week 24 (95% CI −3.4 to 12.5). Primary and sensitivity analyses of the coprimary endpoints are presented in online supplementary figure S1. A post-hoc analysis to determine relative risk is presented in online supplementary table S2.

**Table 2:** Primary efficacy endpoint: estimate and CIs for differences in ACR20 response rate at week 12 and week 24 (FAS)

		n	Proportion (%)	Difference in proportions (BI 695501 – Humira, %)	
				Estimate	CI
Week 12	BI 695501	321	67.0	5.9	90% CI (−0.9 to 12.7)
	Humira	318	61.1		
Week 24	BI 695501	321	69.0	4.5	95% CI (−3.4 to 12.5)
	Humira	318	64.5		

ACR20, American College of Rheumatology 20%; FAS, full analysis set.

**Figure 2:**
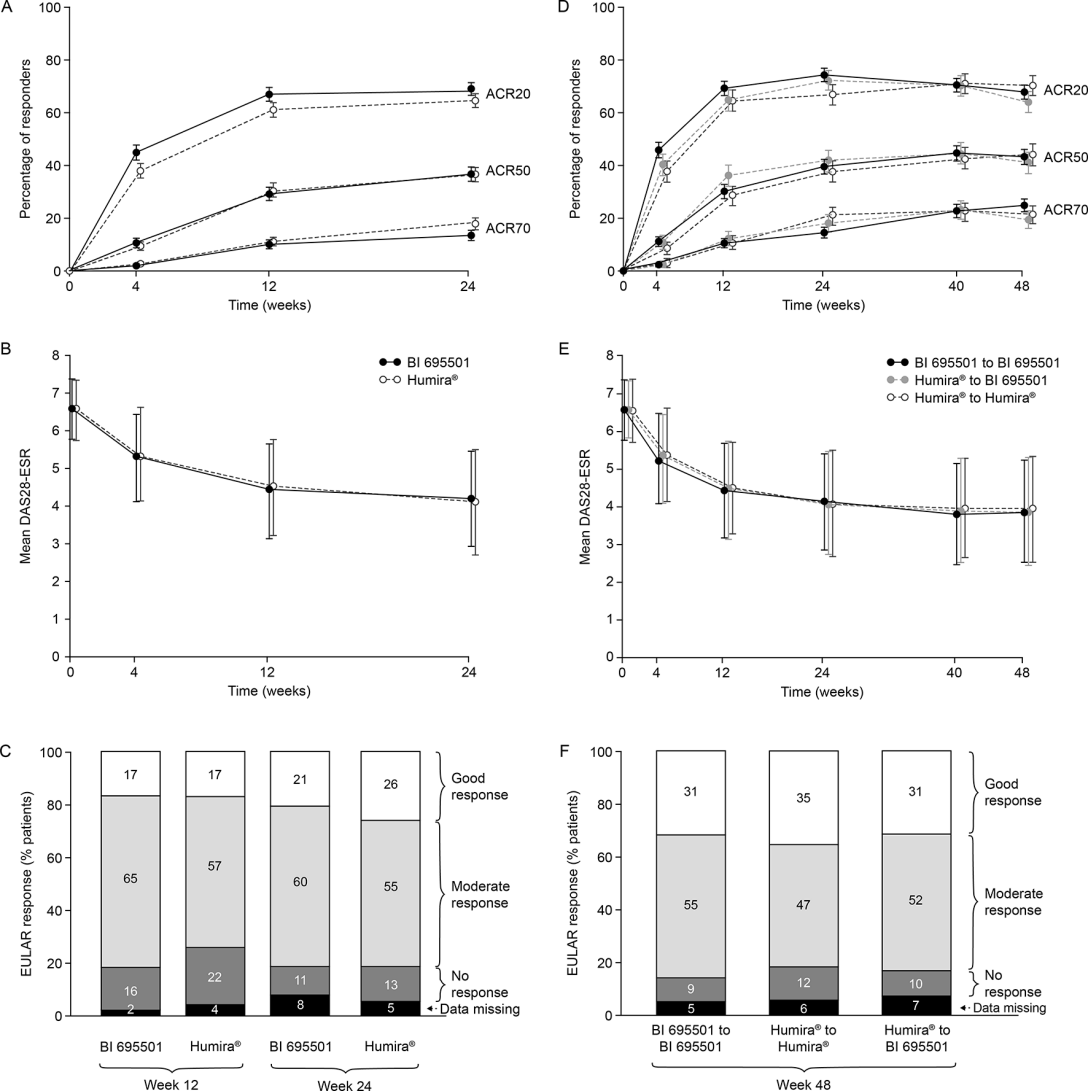
Week 24 results (A–C). Percentage of patients with ACR20/ACR50/ACR70 responses; bars show SEs (A). Mean DAS28-ESR; bars show SDs (B). EULAR responses (C). Week 48 results (D–F). Percentage of patients with ACR20/ACR50/ACR70 responses. Bars show SEs (D). Mean DAS28-ESR; bars show SDs (E). EULAR responses (F). ACR, American College of Rheumatology; DAS28-ESR, Disease Activity Score in 28 joints-erythrocyte sedimentation rate; EULAR, European League Against Rheumatism.

As a sensitivity analysis, the primary efficacy analysis was repeated on the PPS (same imputation methodology; online supplementary figure S1). The similarity of ACR20 responses in the two groups at weeks 12 and 24 was independent of baseline demographic and clinical characteristics (online supplementary figure S2). The analysis of the secondary efficacy endpoints supported the findings of the primary efficacy analysis. The mean percentage of patients meeting the ACR20/50/70 response criteria was similar in each treatment group at weeks 12 and 24 (figure 2A). The mean change from baseline in DAS28-ESR was similar between the two treatment groups at weeks 12 and 24 (online supplementary table S3; figure 2B).

The percentage of patients with European League Against Rheumatism (EULAR)-defined ‘good’ and ‘moderate’ response rates was similar at weeks 12, 24 and 48 (figure 2C; ACR/EULAR Boolean definition^9^ of remission available in online supplementary table S4). Both treatment groups showed a similar increase in the SF-36 physical and mental component scores (weeks 12 and 24), indicating a similar improvement in quality of life (online supplementary figure S3).

#### Results at week 48

Results from baseline to week 48 are presented as per the three treatment groups generated at rerandomisation (week 24). ACR20/50/70 response rates and mean change from baseline in DAS28-ESR were similar across the switched and the continuous groups (figure 2D,E). Similar percentages of patients had ‘good’ and ‘moderate’ EULAR response rates at week 48 in each group (figure 2F).

### Safety

Safety follow-up was to week 58 for all patients who did not enter the OLE. The proportion of patients with drug-related AEs was similar between the treatment groups. Overall, safety findings were similar between the continuous BI 695501 and Humira arms from day 1 to week 58, and between the rerandomised groups from week 24 to 58 (table 3). The frequency of AEs with an incidence of ≥3% to week 58 is presented in online supplementary table S5. Among serious AEs, infections and infestations was the most common system organ class (0.6% for BI 695501 vs 4.0% for Humira). No deaths were reported during the study.

**Table 3:** Overview of AEs (SAF)

Patients with, n (%)	AEs occurring day 1 to week 58			AEs occurring week 24 to week 58		
	BI 695501 to BI 695501 (n=324)	Humira to BI 695501 (n=146)	Humira to Humira (n=175)	BI 695501 to BI 695501 (n=298)	Humira to BI 695501 (n=146)	Humira to Humira (n=148)
At least one AE	193 (59.6)	93 (63.7)	105 (60.0)	126 (42.3)	62 (42.5)	51 (34.5)
At least one drug-related AE	62 (19.1)	28 (19.2)	40 (22.9)	39 (13.1)	17 (11.6)	17 (11.5)
At least one serious AE	18 (5.6)	10 (6.8)	17 (9.7)	6 (2.0)	6 (4.1)	5 (3.4)
At least one serious drug-related AE	2 (0.6)	1 (0.7)	6 (3.4)	1 (0.3)	0 (0.0)	2 (1.4)
AE leading to study drug discontinuation	13 (4.0)	6 (4.1)	12 (6.9)	5 (1.7)	6 (4.1)	1 (0.7)

AE, treatment-emergent adverse event; n, number of patients per group; SAF, safety analysis set.

The most frequently reported AEs leading to drug discontinuation were acute pyelonephritis (n=2) and urticaria (n=2) (both in the Humira group only). Up to week 24, serious infections were pneumonia (n=4), acute pyelonephritis (n=2), and appendicitis, infective arthritis and bronchitis (each, n=1) (Humira group only). Cellulitis was reported for one patient (BI 695501 group). From week 24 to week 58, serious infections were pneumonia in one patient (continuous Humira group), and influenza, viral pneumonia and salmonella sepsis in one patient (Humira to BI 695501 group).

### Immunogenicity

Immunogenicity data were available at week 24 (SAF population; 92.2%; n=595/645) and week 48 (87.8% of total randomised; n=566). At baseline, 32 (5.0%) patients had ADAs against adalimumab (BI 695501 group, n=11; Humira group, n=21). In 25/32 patients these ADAs were neutralising (9 BI 695501; 16 Humira).

**Figure 3:**
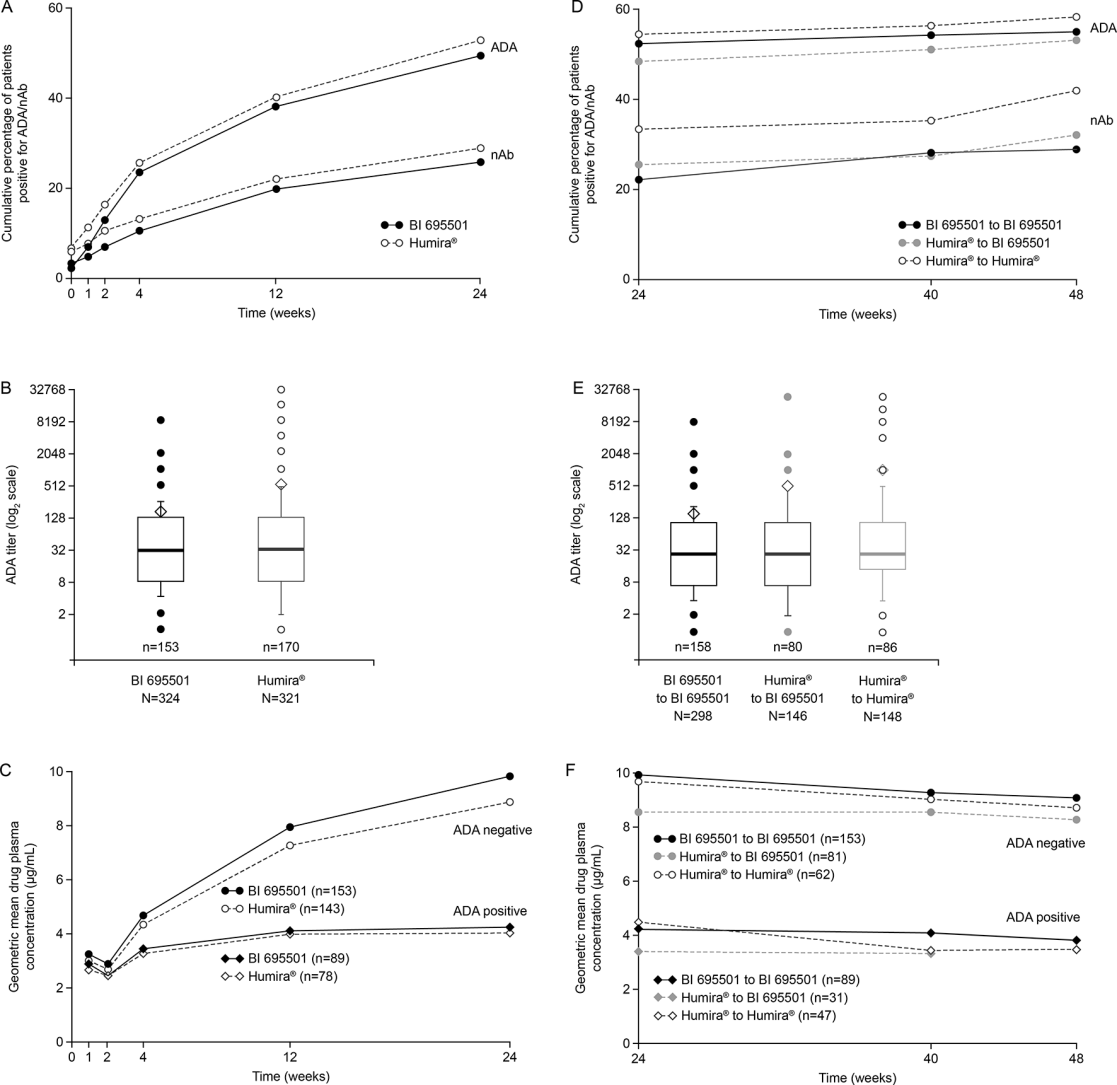
Week 24 results (A–C). Percentage of patients with positive ADA/nAb test (A). ADA titre (B). Drug plasma concentration by presence of ADAs (C). Week 48 results (D–F). Percentage of patients with positive ADA/nAb test (D). ADA titre (E). Drug plasma concentration by presence of ADAs (F). ADA, antidrug antibodies; n, number of patients per group; nAb, neutralising antibodies.

Overall 50.2% of the patients were ADA-positive at any time point up to week 24. The ADA frequencies up to week 24 were similar in the BI 695501 (47.4%) and in the Humira groups (53.0%) (figure 3A). ADA titres at week 24 (figure 3B) and nAb frequencies up to week 24 (figure 3A) were also similar between the groups. Whether or not patients transitioned from Humira to BI 695501 or continued on Humira did not influence subsequent ADA frequency and titres. Similar immunogenicity was observed after week 24 in all rerandomised groups (ADA-positive patients at any time point up to week 48, figure 3D; nAb-positive patients at any time point up to week 48, figure 3D; ADA titres at week 48, figure 3E).

The impact of ADA on drug plasma levels at weeks 24 and 48 is shown in figure 3C,F. A lower drug concentration was measured in ADA-positive patients compared with ADA-negative patients. This effect was independent of treatment group. Overall, in a post-hoc exploratory analysis, ADA-positive patients had a numerically slightly lower median change from baseline in DAS28-CRP at week 48 than ADA-negative patients (week 48: ADA-positive −2.25, ADA-negative −2.52).

## Discussion

The efficacy, safety and immunogenicity results of this study demonstrate clinical equivalence of BI 695501 and Humira. In combination with the phase I VOLTAIRE-PK study data^6^ plus prior physicochemical and functional analyses (manuscripts in preparation), VOLTAIRE-RA completes the similarity assessment of the adalimumab biosimilar BI 695501.

### Selection of study design and endpoints

A biosimilar phase III study needs to be designed to optimise the chance of detecting potential clinical differences between the biosimilar candidate and reference product.^1 2^ A suitable clinical model is characterised by a combination of certain factors, including a disease and a population that will respond to the treatment with a large effect size and endpoints that are sensitive to measure those responses. A homogeneous population increases the sensitivity to detect differences because of the reduction of interindividual variability. The clinical model is usually selected in consultation with regulatory agencies.

Adalimumab is a standard of care in a wide range of autoimmune diseases and is commonly used in adults and children. Phase III studies for other adalimumab biosimilar candidate molecules have been completed or are ongoing in adult patients with RA (eg, with SB5,^10^ FKB327^11^ and PF-06410293^12^) or plaque psoriasis (including with MYL-1401A,^13^ CHS-1420,^14^ M923^15^ and MSB11022^16^) or both (ABP 501^17 18^ and GP2017^19 20^). RA was selected for the main phase III study with BI 695501 due to its sensitivity to adalimumab, the availability of ACR20 as a well-established and sensitive measure of disease activity reduction, and operational feasibility. Supportive phase III studies are ongoing with BI 695501 in plaque psoriasis (NCT02850965) and Crohn’s disease (NCT02871635).

### Switching from reference product Humira to BI 695501

When switching from the reference product to a biosimilar for which clinical similarity has been established (as presented here for Humira to BI 695501), one would not expect a change in efficacy or safety, although natural fluctuation of disease activity in individual patients may occur. It is important that such treatment switches are studied and understood as this may become common practice within routine care (eg, many patients transitioned from Remicade to its biosimilars as they became available).

Here, patients who had been treated with Humira (baseline–24 weeks) were randomised to either continue Humira or switch to BI 695501 (weeks 25–48). No differences were detected between these two groups with regard to adherence to treatment, efficacy, safety and immunogenicity. Future, observational studies could help confirm these findings in the real-world setting. Since biosimilars to Remicade have become available, a number of observational and interventional studies (such as the NOR-SWITCH study)^21^ have confirmed the findings of the development programme of CT-P13 (Remsima, Inflectra), suggesting that developing biosimilars in a programme that relies on analytical, preclinical and limited clinical studies is a robust concept.

### Overall assessment of efficacy and safety

The efficacy data from VOLTAIRE-RA indicate that BI 695501 and Humira have therapeutic equivalence at week 12 and week 24. Up to week 48, the mean change from baseline in DAS28-ESR and ACR20/50/70 response rates was similar across the switched and continuous groups. BI 695501 and Humira demonstrated similar safety and tolerability; there were no new safety findings for adalimumab. The frequency of hypersensitivity or injection site reactions was similarly frequent in all treatment groups.

### Overall assessment of immunogenicity

Immunogenicity is a key aspect of the clinical similarity evaluation of a biosimilar agent. Therefore, a highly sensitive and drug-tolerant ADA assay was developed and applied during clinical development of BI 695501. Different and often less sensitive ADA and nAb assays were used in historical trials (eg, pivotal trials with Humira^22^). This can explain previous reports of different frequencies of ADA-positive and nAb-positive patients detected in earlier studies. Overall, similar immunogenicity (ADA frequency and titres, and nAb frequency) was observed between BI 695501 and Humira throughout this study. Patients switching from Humira to BI 695501 did not demonstrate increased immunogenicity or more hypersensitivity reactions compared with patients continuing to receive Humira.

As expected, an inverse correlation between ADAs and drug plasma concentration was detected. This effect was similar between the BI 695501 and Humira at week 24 and between the three study groups at week 48. This confirms previous data from the VOLTAIRE-PK study^6^ showing a comparable impact of ADA on key pharmacokinetic parameters for BI 695501, and USA-approved and EU-approved Humira. An explanation of the pre-existence of antibodies in otherwise drug-naïve subjects is given in online supplementary appendix D.

### Adalimumab biosimilar landscape

The introduction of adalimumab was a major step forward for patients suffering from certain chronic immune-mediated diseases. Its benefits for patients, along with the prevalence of its indications, led to healthcare system costs exceeding US$15 billion (2016). It is therefore unsurprising that several companies are developing biosimilars to Humira. Currently, Amgen’s ABP 501 (Amgevita/Amjevita) is FDA-approved and EMA-approved.^22 23^ Clinical study results are typically similar for different biosimilar candidates due to comparable study designs (regulator agency requirement) and inclusion of AbbVie’s reference product Humira as the common comparator.

## Conclusion

VOLTAIRE-RA showed that BI 695501 and Humira are highly similar in terms of efficacy, safety and immunogenicity. The switch from Humira to BI 695501 had no impact on efficacy, safety and immunogenicity. These data, together with the analytical and the phase I data, suggest that BI 695501 and Humira are biosimilar and thus therapeutically equivalent.

## References

[1] Food and Drug Administration. Scientific considerations in demonstrating biosimilarity to a reference product. 2012 https://www.fda.gov/downloads/DrugsGuidanceComplianceRegulatoryInformation/Guidances/UCM291128.pdf

[2] European Medicines Agency. Guideline on similar biological medicinal products. 2014 http://www.ema.europa.eu/docs/en_GB/document_library/Scientific_guideline/2014/10/WC500176768.pdf

[3] European Medicines Agency. Guideline on similar biological medicinal products containing monoclonal antibodies - non-clinical and clinical issues. 2012 http://www.ema.europa.eu/docs/en_GB/document_library/Scientific_guideline/2012/06/WC500128686.pdf

[4] Hoffman JM, Li E, Doloresco F, et al Projecting future drug expenditures--2012. Am J Health Syst Pharm 2012;69:405–21. 10.2146/ajhp110697 22345420

[5] Scott DL, Cope A New tumour necrosis factor inhibitors for rheumatoid arthritis: are there benefits from extending choice? Ann Rheum Dis 2009;68:767–9. 10.1136/ard.2008.105940 19435722

[6] Wynne C, Altendorfer M, Sonderegger I, et al Bioequivalence, safety and immunogenicity of BI 695501, an adalimumab biosimilar candidate, compared with the reference biologic in a randomized, double-blind, active comparator phase I clinical study (VOLTAIRE®-PK) in healthy subjects. Expert Opin Investig Drugs 2016;25:1361–70. 10.1080/13543784.2016.1255724 27813422

[7] Reeve R Confidence interval of difference of proportions in logistic regression in presence of covariates. Stat Methods Med Res 2018;27:451–65. 10.1177/0962280216631583 26988932

[8] Rubin DB Multiple imputation for nonresponse in surveys. New Jersey, USA: John Wiley & Sons, Inc, 1987:1–26.

[9] Felson DT, Smolen JS, Wells G, et al American College of Rheumatology/European League against rheumatism provisional definition of remission in rheumatoid arthritis for clinical trials. Arthritis Rheum 2011;63:573–86. 10.1002/art.30129 21294106 PMC3115717

[10] Clinicaltrials.Gov. A study comparing SB5 to Humira in subjects with moderate to severe rheumatoid arthritis despite methotrexate therapy. 2017 NCT02167139 https://clinicaltrials.gov/ct2/show/results/NCT02167139?term=sb5&rank=2&sect=X70156

[11] Clinicaltrials.Gov. A study to compare FKB327 efficacy and safety with humira in rheumatoid arthritis patients (ARABESC). 2017 NCT02260791 https://clinicaltrials.gov/ct2/show/NCT02260791

[12] Clinicaltrials.Gov. A Study of PF-06410293 (Adalimumab-Pfizer) and Adalimumab (Humira) in Combination With Methotrexate In Subjects With Active Rheumatoid Arthritis (REFLECTIONS B538-02). 2017 NCT02480153.

[13] Clinicaltrials.Gov. MYL-1401A Efficacy and safety comparability study to humira. 2017 NCT02714322 https://clinicaltrials.gov/ct2/show/NCT02714322

[14] Clinicaltrials.Gov. Comparison of CHS-1420 Versus Humira in subjects with chronic plaque psoriasis (PsOsim). 2017 NCT02489227 https://clinicaltrials.gov/ct2/show/NCT02489227

[15] Clinicaltrials.Gov. Phase 2 Study of M923 and Humira® in Subjects With Chronic Plaque-type Psoriasis. 2017 NCT02581345 https://clinicaltrials.gov/ct2/show/NCT02581345

[16] Clinicaltrials.Gov. MSB11022 in Moderate to severe chronic plaque psoriasis (AURIEL-PsO). 2017 NCT02660580 https://clinicaltrials.gov/ct2/show/NCT02660580

[17] Clinicaltrials.Gov. Efficacy and Safety Study of ABP 501 Compared to Adalimumab in Subjects With Moderate to Severe Rheumatoid Arthritis. 2017 NCT01970475 https://clinicaltrials.gov/ct2/show/NCT01970475 10.1136/annrheumdis-2016-210459 PMC5629940 28584187

[18] Clinicaltrials.Gov. Study to Compare Efficacy and Safety of ABP 501 and Adalimumab (HUMIRA®) in Adults With Moderate to Severe Plaque Psoriasis. 2017 NCT01970488 https://clinicaltrials.gov/ct2/show/NCT01970488

[19] Clinicaltrials.Gov. Clinical Trial to Compare Treatment With GP2017 and Humira® in patients with rheumatoid arthritis (ADMYRA). 2017 NCT02744755 https://clinicaltrials.gov/ct2/show/NCT02744755

[20] Clinicaltrials.Gov.. Study to Demonstrate Equivalent Efficacy and to Compare Safety to Biosimilar Adalimumab (GP2017) and Humira (ADACCESS). 2017 NCT02016105 https://clinicaltrials.gov/ct2/show/NCT02016105

[21] Jørgensen KK, Olsen IC, Goll GL, et al Switching from originator infliximab to biosimilar CT-P13 compared with maintained treatment with originator infliximab (NOR-SWITCH): a 52-week, randomised, double-blind, non-inferiority trial. Lancet 2017;389:2304–16. 10.1016/S0140-6736(17)30068-5 28502609

[22] Abbvie Limited. Humira 40 mg/0.4 ml Pre-filled Syringe and Pre-filled Pen. Summary of Product Characteristics. 2017 https://www.medicines.org.uk/emc/medicine/31860

[23] Food and Drug Administration. FDA approved Amjevita, a biosimilar to Humira®. 2016 https://www.fda.gov/newsevents/newsroom/pressannouncements/ucm522243.html

