# Supplementary material for: Similar efficacy, safety and immunogenicity of adalimumab biosimilar BI 695501 and Humira reference product in patients with moderately to severely active rheumatoid arthritis: results from the phase III randomised VOLTAIRE-RA equivalence study
Source: Ann Rheum Dis. 2018 Mar 7;77(6):914–21. doi: 10.1136/annrheumdis-2017-212245 (PMC5965346; doi:10.1136/annrheumdis-2017-212245)
Supplement: Supplementary data [file annrheumdis-2017-212245supp001.docx]

**Supplementary Information**

- **Appendix A** Randomisation procedure
- **Appendix B** Inclusion and exclusion criteria
- **Appendix C** Immunogenicity assessment
- **Appendix D** Pre-existence of antibodies to drug-naïve subjects
- **Figure S1** All primary and sensitivity analyses of the co-primary end points
- **Figure S2** Subgroup analyses of ACR20 response by baseline demographic and clinical characteristics at (A) week 12 (A) and week 24 (B) for the full analysis set
- **Figure S3** Change in SF-36 mental and physical component scores at week 12 and 24 for the full analysis set
- **Table S1** Patient disposition: geographic location
- Table S2 Primary efficacy: Estimate and confidence intervals for relative risk in ACR20 response rate at week 12 and week 24 (FAS)
- **Table S3** Secondary efficacy end points: ANCOVA models for DAS28-ESR at week 12 and week 24 (FAS)
- **Table S4** Achievement of remission as per ACR/EULAR Boolean definition
- **Table S5** Frequency of AEs with an incidence of ≥3% by system organ class and preferred term based on the preferred term level up to week 58 (SAF)

**Appendix A. Randomisation procedure**

Randomisation was performed using an IRT system (Almac Clinical Technologies, Souderton, PA, USA) and was stratified according to region (Asia, EU, Latin America, US) and prior exposure to a biologic agent (yes/no). Randomisation was achieved through a pre-randomisation call or through a standard randomisation visit call on day 1 in block size of 4, and patients were randomised sequentially (i.e. the lowest sequentially available randomisation number). Investigators and trial personnel remained blinded with regard to the randomised treatment assignments until after database lock. Unblinded trial personnel who received and handled the trial drug were not involved in any other trial assessments or procedures and did not interact with patients in any way.

Re-randomisation was performed in a similar way with stratification being limited to prior exposure to one biologic without stratification for region.

**Appendix B. Inclusion and exclusion criteria**

**Inclusion criteria**

Patients were eligible for inclusion if they fulfilled each of the following criteria:

1. All patients had to sign and date an informed consent form consistent with International Council for Harmonisation Good Clinical Practice guidelines and local legislation prior to participation in the trial (i.e. prior to any trial procedures, which included medication washout and restrictions) and had to be willing to follow the protocol.
2. Male or female participants, between 18 and 80 years of age, with a diagnosis of moderately to severely active rheumatoid arthritis (RA) for at least 6 months as defined by at least six swollen joints (66 joint count) and at least six tender joints (68 joint count) at screening and baseline (day 1), and either an ESR of >28 mm/hour **OR** a C-reactive protein (CRP) level >1.0 mg/dl (normal: <0.4 mg/dl) at screening. Patients must also have been receiving MTX therapy.
3. Current treatment for RA on an outpatient basis:
4. Must have been receiving and tolerating oral or parenteral MTX therapy at a dose of 15 to 25 mg per week (dose may have been as low as 10 mg per week if the patient was unable to tolerate a higher dose) for at least 12 weeks immediately prior to day 1. The dose and administration route had to remain stable for at least 4 weeks prior to day 1 until week 24. After week 24, the administration route could have been changed at the Investigator’s discretion. Patients receiving a lower dose of MTX (10 to 14 mg/week) should have been doing so as a result of a documented history of intolerance to higher doses of MTX.
5. Patients had to be willing to receive oral folic acid (at least 5 mg/week or as per local practice) or folinic acid (at least 1 mg/week or as per local practice) or equivalent during the entire trial (mandatory co-medication for MTX treatment).
6. Disease-modifying anti-rheumatic drug (DMARD) use was restricted according to guidelines listed in the study protocol.
7. If receiving current treatment with oral corticosteroids (other than intra-articular or parenteral), the dose must not have exceeded 10 mg/day prednisolone or equivalent. During the 4 weeks prior to baseline (day 1), the dose had to remain stable.
8. Any concomitant non-steroidal anti-inflammatory drugs (NSAIDs) had to be stable for at least 2 weeks prior to day 1.
9. Patients could have been taking oral hydroxychloroquine provided that the dose was not greater than 400 mg/day or chloroquine provided that the dose was not greater than 250 mg/day. These doses must have been stable for a minimum of 12 weeks prior to day 1. The hydroxychloroquine or chloroquine treatment had to be continued at a stable dose with the same formulation until the end of the trial.
10. For participants of reproductive potential (males and females), a reliable means of contraception had to be used throughout trial participation. Acceptable methods of birth control included, for example, birth control pills, intrauterine devices (IUDs), surgical sterilisation, vasectomised partner and double-barrier method (for example male condom in combination with female diaphragm/cervical cap plus spermicidal foam/gel/film/cream/suppository). All patients (males and females of child-bearing potential) also had to agree to use an acceptable method of contraception for 6 months following completion or discontinuation from the trial drug.

**Exclusion criteria**

Patients were to be excluded if they met any of the following criteria:

1. American College of Rheumatology (ACR) functional Class IV or wheelchair/bed bound.
2. Primary or secondary immunodeficiency (history of, or currently active), including known history of HIV infection, or a positive test at screening (per the Investigator discretion and/or where mandated by local authorities.
3. History of tuberculosis (TB), latent TB, or positive purified protein derivative (PPD) test or interferon gamma-releasing assay (IGRA).
4. Known clinically significant coronary artery disease or significant cardiac arrhythmias or severe congestive heart failure (New York Heart Association Classes III or IV), or interstitial lung disease observed on chest X-ray.
5. Previous treatment with ≥2 biologic agents. Patients who had received prior treatment with 1 biologic agent > 4 months prior to screening may have participated in the trial.
6. Previous treatment with adalimumab or adalimumab biosimilar.
7. Current treatment or previous treatment with leflunomide within 8 weeks (56 days) prior to day 1.
8. History of a severe allergic reaction or anaphylactic reaction to a biological agent or history of hypersensitivity to adalimumab or any component of the trial drug.
9. History of cancer including solid tumours, haematological malignancies and carcinoma in situ (except participants with previous resected and cured basal or squamous cell carcinoma, treated cervical dysplasia, or treated in situ grade 1 cervical cancer within 5 years prior to the screening visit).
10. Evidence of positive serology for hepatitis B virus (HBV) or hepatitis C virus (HCV).
11. Receipt of a live/attenuated vaccine within 12 weeks prior to the screening visit. Patients who were expecting to receive any live virus or bacterial vaccinations during the trial, or up to 3 months after the last dose of trial drug.
12. Any treatment (including biologic therapies) that, in the opinion of the Investigator, may have placed the patient at unacceptable risk during the trial.
13. A significant disease other than RA and/or a significant uncontrolled disease (such as, but not limited to, nervous system, renal, hepatic, endocrines or gastrointestinal disorders). A significant disease was defined as a disease which, in the opinion of the Investigator, may (i) have put the patient at risk because of participation in the trial, or (ii) have influenced the results of the trial, or (iii) have caused concern regarding the patient's ability to participate in the trial.
14. Premenopausal (last menstruation 1 year prior to screening), sexually active women who were pregnant or nursing, or were of child-bearing potential and not practicing an acceptable method of birth control or did not plan to continue practicing an acceptable method of birth control throughout the trial (acceptable methods of birth control were IUDs, surgical sterilisation, double barrier, or vasectomised partner).
15. History of, or current, inflammatory joint disease other than RA (e.g. gout, reactive arthritis, psoriatic arthritis, seronegative spondyloarthropathy and Lyme disease) or other systemic autoimmune disorder (e.g. systemic lupus erythematosus, inflammatory bowel disease, pulmonary fibrosis, or Felty’s syndrome, scleroderma, inflammatory myopathy, mixed connective tissue disease or any overlap syndrome). Secondary Sjögren’s syndrome or secondary limited cutaneous vasculitis with RA was permitted.
16. Diagnosis of juvenile idiopathic arthritis, also known as juvenile RA, and/or RA before 16 years of age.
17. Any planned surgical procedure, including bone/joint surgery/synovectomy (including joint fusion or replacement) within 12 weeks prior to the screening visit or for the duration of the trial.
18. Known active infection of any kind (excluding fungal infections of nail beds), or any major episode of infection requiring hospitalisation or treatment with intravenous anti-infectives within 4 weeks of the screening visit or completion of oral anti-infectives within 2 weeks of the screening visit.
19. History of deep space/tissue infection (e.g. fasciitis, abscess, osteomyelitis, native or prosthetic joint infection) within 52 weeks of the screening visit.
20. History of serious infection or opportunistic infection in the last 2 years (to screen for a chest infection, a chest X-ray was to be performed at screening if one was not performed within 12 weeks of the screening visit).
21. Any neurological (congenital or acquired), vascular or systemic disorder that might have affected any of the efficacy assessments, in particular, joint pain and swelling (e.g. Parkinson’s disease, cerebral palsy and diabetic neuropathy).
22. Currently active alcohol or drug abuse or history of alcohol or drug abuse (as determined by the Investigator) within 2 years of the screening visit.
23. Treatment with intravenous Gamma Globulin or the Prosorba® Column within 6 months of the screening visit.
24. Treatment with intravenous, intramuscular, intra-articular and parenteral corticosteroids within 6 weeks prior to day 1 or throughout the trial.
25. Aspartate aminotransferase (AST) or alanine aminotransferase (ALT) >1.5 x upper limit of normal (ULN).
26. Haemoglobin <8.0 g/dl.
27. Platelets <100,000/μl.
28. Leukocyte count <4,000/μl.
29. Creatinine clearance <60 ml/min.
30. Current or prior participation in another clinical trial (inclusive of follow-up/administrative visits) with another investigational drug within a minimum of 12 weeks or five half-lives (whichever is longer) of the drug prior to day 1. Previous randomisation in this trial.
31. A history of any clinically significant adverse reaction to murine or chimeric proteins, including serious allergic reactions.

**Appendix C. Immunogenicity assessment**

Immunogenicity evaluations were performed on the safety population as previously described (Wynne C, et al., Expert Opin Invest Drugs 2016;25:1361–70). Blood samples for ADA and nAb analysis were taken pre-dose and at weeks 1, 2, 4, 12, 24, 40, 48 (end of treatment) and 58 (end of study). ADAs against BI 695501 and Humira® were measured using a sequential approach, applying a screening, confirmatory and titer assay. ADA-status and titer were determined using a bridging electrochemiluminescence assay based on BI 695501-labeled reagents. The sensitivity of the ADA assay was set at 4.7 ng/ml and the drug tolerance was 30 µg/ml (free drug concentration). Since the maximal measured drug concentration in this study was <25 µg/ml, no drug interference in the ADA measurements was expected. Identification of nAbs against BI 695501 and Humira® was conducted using a cell-based, antibody-dependent cell-mediated cytotoxicity method. Assay methodologies were fully validated in accordance with International Council for Harmonisation guidelines.

**Appendix D. Pre-existence of antibodies to drug-naïve subjects**

The pre-existence of antibodies in otherwise drug-naïve subjects (5% in this study) is a well-described phenomenon (Xue L, et al., *AAPS Journal* 2013;15:852–5). In a recently published equivalence study of ABP 501, it was shown that 11 of the 526 patients (2.1%) tested positive for pre-existing antibodies (Cohen S, et al., *Ann Rheum Dis* 2017;76:1679–87); a similar incidence to this study. Gorovits et al. (Gorovits B, et al., *AAPS Journal* 2016;18:311–20) describe three major matrix components that can lead to reactivity in ADA assays: endogenous proteins that can bind to the drug, e.g., multimeric drug targets (thought unlikely here; during assay development up to 50 ng/ml of TNFα did not cause an ADA false-positive response), non-specific interfering agents such as rheumatoid factor (RF) (excluded here; during validation it was shown that RF did not interfere with the assay’s ability to confirm positive and negative samples up to a concentration of 10,000 IU/mL) and naturally occurring antibodies to proteins and glycans cross-reactive with drug-specific epitopes. The latter cannot be fully excluded as the reason for pre-dose positive ADA results. However, potential cross-reacting antibodies did not have any impact on the immunogenicity of adalimumab or the impact of the immunogenic response on PK parameters or safety events.

**Figure S1** All primary and sensitivity analyses of the co-primary end points.

The 90% CI for the difference in the percentage (BI 695501– Humira®) of patients achieving an ACR20 response at week 12, and the 95% CI for the same difference at week 24 in the PPS was contained within the same pre-specified interval as the primary analysis (−12%; 15%).


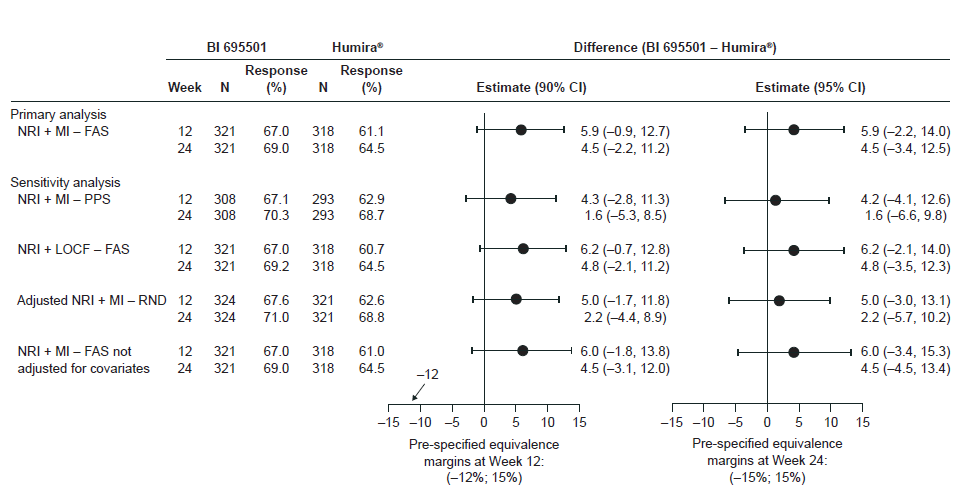


ACR20, American College of Rheumatology 20% response criteria; CI, confidence interval; FAS, full analysis set; LOCF, last observation carried forward; MI, multiple imputation; N, number of patients per group; NRI, non-responder imputation; PPS, per-protocol analysis set; RND, all patients randomised analysis set**.**

**Figure S2** Subgroup analyses of ACR20 response by baseline demographic and clinical characteristics at (A) week 12 (A) and week 24 (B) for the full analysis set


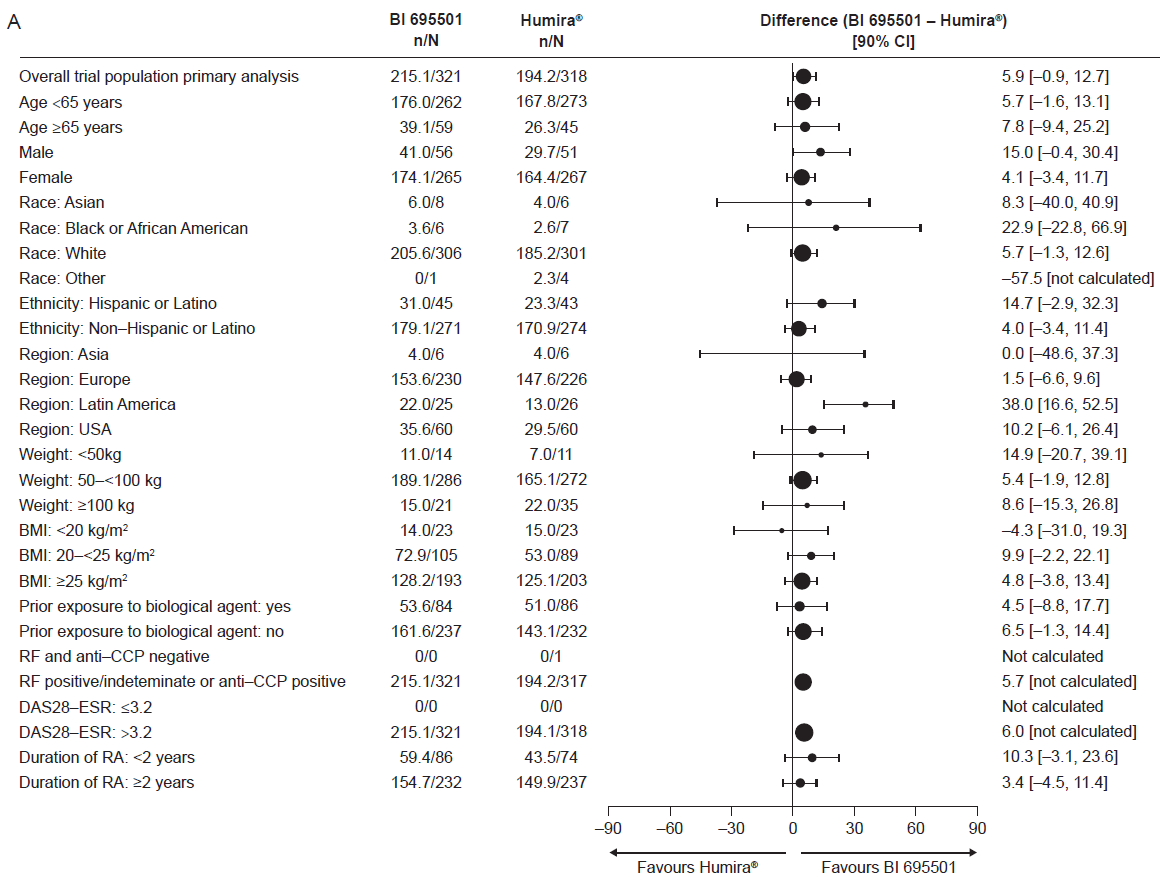


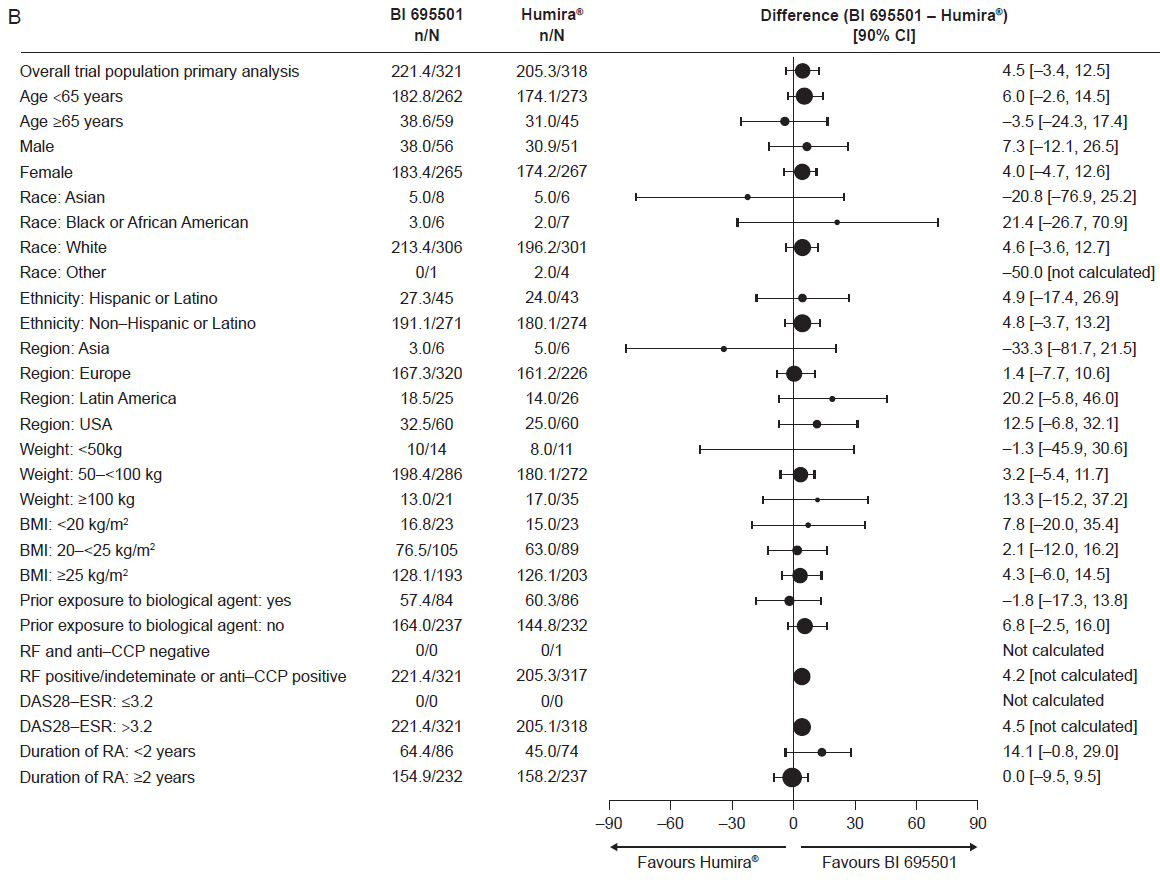


ACR20, American College of Rheumatology 20% response criteria; BMI, body mass index; CCP, cyclic citrullinated peptide; CI, confidence interval; DAS28-ESR, Disease Activity Score 28-joint count erythrocyte sedimentation rate; N, number of patients per group; RA, rheumatoid arthritis; RF, rheumatoid factor.


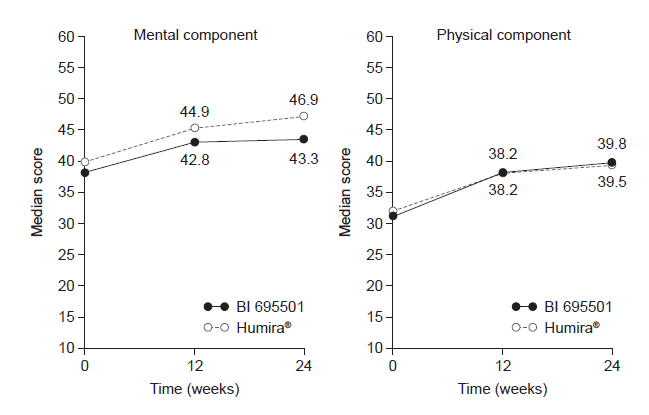
**Figure S3** Change in SF-36 mental and physical component scores at week 12 and 24 for the full analysis set

SF-36, 36-Item Short-Form Health Survey.

| **Table S1** Patient disposition: geographic location | |
| --- | --- |
| Country | Patients (%) |
| Poland | 25.0 |
| United States | 19.1 |
| Ukraine | 17.1 |
| Bulgaria | 10.7 |
| Chile | 7.9 |
| Serbia | 5.0 |
| Russian Federation | 4.2 |
| Hungary | 4.0 |
| Spain | 1.9 |
| Germany | 1.7 |
| Estonia | 1.7 |
| Republic of Korea | 0.8 |
| Thailand | 0.8 |
| Malaysia | 0.3 |

| **Table S2** Primary efficacy: Estimate and confidence intervals for relative risk in ACR20 response rate at week 12 and week 24 (FAS) | | | | |
| --- | --- | --- | --- | --- |
|  | | **Relative risk** | | |
|  | | **n** | **Estimate** | **Confidence interval** |
| Week 12 | BI 695501 | 321 | 1.10 | 90% CI: (1.00 to 1.21)  95% CI: (0.96 to 1.19) |
|  | Humira® | 318 |  |  |
| Week 24 | BI 695501 | 321 | 1.07 |  |
|  | Humira® | 318 |  |  |

ACR20, American College of Rheumatology 20% response criteria; CI, confidence interval; Estimate, relative risk in ACR20 response rate between BI 695501 and Humira®; N, number of patients per group.

| **Table S3** Secondary efficacy end points: ANCOVA models for DAS28-ESR at week 12 and week 24 (FAS) | | | | | | |
| --- | --- | --- | --- | --- | --- | --- |
|  | |  | **Mean change from baseline** | | **Treatment difference  (BI 695501 – Humira®)** | |
|  | | **n** | **LS Mean** | **95% CI** | **LS Mean** | **95% CI** |
| Week 12 | BI 695501 | 319.6 | -2.1 | (-2.28 to -2.01) |  |  |
|  | Humira® | 317.1 | -2.0 | (-2.18 to -1.91) | -0.1 | (-0.28 to 0.08) |
| Week 24 | BI 695501 | 313.9 | -2.4 | (-2.51 to -2.21) |  |  |
|  | Humira® | 315.1 | -2.4 | (-2.54 to -2.24) | 0 | (-0.17 to 0.23) |

ANCOVA, analysis of covariance; CI, confidence interval; DAS28-ESR, Disease Activity Score 28-joint count erythrocyte sedimentation rate; FAS, full analysis set; LS, least squares; N, number of patients per group.

| **Table S4** Achievement of remission as per ACR/EULAR Boolean definition^1^ | | |
| --- | --- | --- |
| n (%) | | **ACR/EULAR remission**  **(Boolean definition)** |
| Week 12 | BI 695501 | 6 (1.9) |
|  | Humira® | 1 (0.3) |
| Week 24 | BI 695501 | 6 (1.9) |
|  | Humira® | 3 (0.9) |

ACR, American College of Rheumatology; EULAR, European League Against Rheumatism; N, number of patients per group.

1. Felson DT, Smolen JS, Wells G, et al. American College of Rheumatology/European League Against Rheumatism provisional definition of remission in rheumatoid arthritis for clinical trials. Arthritis Rheum 2011;63:573–86.

| **Table S5** Frequency of AEs with an incidence of ≥3% by system organ class and preferred term based on the preferred term level up to week 58 (SAF) | | |
| --- | --- | --- |
| **Patients with, n (%)** | **BI 695501**  **to**  **BI 695501**  **(n=324)** | **Humira®**  **to**  **Humira®**  **(n=175)** |
| At least one AE | 193 (59.6) | 105 (60.0) |
| Infections and infestations | 114 (35.2) | 60 (34.3) |
| Upper respiratory tract infection | 17 (5.2) | 10 (5.7) |
| Nasopharyngitis | 19 (5.9) | 17 (9.7) |
| Bronchitis | 15 (4.6) | 10 (5.7) |
| Pharyngitis | 13 (4.0) | 5 (2.9) |
| Sinusitis | 10 (3.1) | 6 (3.4) |
| Urinary tract infection | 10 (3.1) | 4 (2.3) |
| Gastroenteritis | 10 (3.1) | 2 (1.1) |
| Nervous system disorders | 23 (7.1) | 12 (6.9) |
| Headache | 11 (3.4) | 6 (3.4) |
| Musculoskeletal and connective tissue disorders | 39 (12.0) | 20 (11.4) |
| Rheumatoid arthritis | 12 (3.7) | 6 (3.4) |
| Investigations* | 31 (9.6) | 11 (6.3) |
| ALT increased | 10 (3.1) | 4 (2.3) |

AE, treatment-emergent adverse event; ALT, alanine aminotransferase; N, number of patients per group; SAF, safety analysis set.

*Laboratory tests and other medical investigations that gave an unusual reading.
